# FORTIFYING DEMENTIA CARE: EXPLORING FAMILY SELF-EFFICACY IN DECISION-MAKING, TRUST, AND QUALITY

**DOI:** 10.1093/geroni/igae098.1763

**Published:** 2024-12-31

**Authors:** Ruth Lopez, Dalit Zaguri-Greener, Lola Kolpina, Anna Zisberg

**Affiliations:** MGH Institute of Health Professions, Boston, Massachusetts, United States; University of Haifa, Haifa, Hefa, Israel; University of Haifa, Haifa, Hefa, Israel; University of Haifa, Haifa, Hefa, Israel

## Abstract

In Israel and many countries, individuals with advanced dementia often spend their final days in nursing homes (NHs). Families prioritize comfort, yet residents often endure significant symptom burden. This discrepancy arises partly because NH staff depend on families to guide end-of-life care. However, family members often lack confidence as decision-makers and struggle to partner with NH staff to translate their intimate knowledge of their loved ones’ preferences into comfort-focused strategies. The purpose of this pilot study employing cross sectional survey design, was to explore relationships among family self-efficacy for surrogate decision-making, trust with staff, and quality of care measures. Among 66 family members of NH residents with advanced dementia in Israel (average age 60, 53% female) self-efficacy was positively associated with trust (r=.32, p<.001), quality of decision-making (r=.34, p<.001), communication (r=.33, p<.001), family-staff relationships (r=.30, p<.01), basic care, and symptom management (r =.36, p<.001). Recognizing the pivotal role of family self-efficacy, interventions to enhance it may be warranted. Our findings also urge further investigation into the intricate interplay between these factors and the need to explore the directionality of these relationships. Understanding the influence of broader contextual factors, such as overall NH care quality, is crucial. Our study establishes a foundation for future inquiries, fostering a comprehensive understanding to enhance the well-being and advanced comfort of individuals with advanced dementia in NH settings.

